# Interprofessional education on complex patients in nursing homes: a focus group study

**DOI:** 10.1186/s12909-021-02867-6

**Published:** 2021-09-24

**Authors:** K. Svensberg, B. G. Kalleberg, E. O. Rosvold, L. Mathiesen, H. Wøien, L. H. Hove, R. Andersen, T. Waaktaar, H. Schultz, N. Sveaass, R. Hellesö

**Affiliations:** 1 Section for Pharmaceutics and Social Pharmacy, Department of Pharmacy, University of Oslo, Oslo, Norway; 2 Department of Pharmacy, Faculty of Pharmacy, University of Uppsala, P.O. Box 580, 751 23 Uppsala, Sweden; 3 Faculty of Medicine, University of Oslo, P.O. 1078, 0316 Blindern, Oslo Norway; 4 Section for Pharmacology and Pharmaceutical Biosciences, Department of Pharmacy, University of Oslo, P.O. 1068, 0316 Blindern, Oslo Norway; 5 Department of Nursing Science, Institute of Health and Society, Faculty of Medicine, University of Oslo, P.O. 1130, 0318 Blindern, Oslo Norway; 6 Institute of Clinical Dentistry, Faculty of Dentistry, University of Oslo, P.O 1109, 0317 Blindern, Oslo Norway; 7 Institute of Health and Society, Faculty of Medicine, University of Oslo, P.O. 1130, 0318 Blindern, Oslo Norway; 8 Department of Psychology, University of Oslo, P.O. 1094, 0317 Blindern, Oslo Norway; 9 Department of Pharmacy, University of Oslo, P.O. 1068, 0316 Blindern, Oslo Norway

**Keywords:** Interprofessional education, Graduate students, Workplace learning, Nursing homes, Focus groups, Complex patients

## Abstract

**Background:**

An ageing population leads up to increasing multi-morbidity and polypharmacy. This demands a comprehensive and interprofessional approach in meeting patients’ complex needs. This study describes graduate students’ experiences of working practice based in interprofessional teams with complex patients’ care needs in nursing homes.

**Method:**

Students from advanced geriatric nursing, clinical nutrition, dentistry, medicine and pharmacy at the University of Oslo in Norway were assigned to groups to examine and develop a care plan for a nursing home patient during a course. Focus groups were used, 21 graduate students participating in four groups. Data were collected during spring 2018, were inductively analysed according to a thematic analysis method (Systematic Text Condensation). An analytical framework of co-ordination practices was applied to get an in-depth understanding of the data.

**Results:**

Three themes were identified: 1) Complex patients as learning opportunities*-* an eye-opener for future interprofessional collaboration 2) A cobweb of relations, and 3) Structural facilitators for new collective knowledge*.* Graduate university students experienced interprofessional education (IPE) on complex patients in nursing homes as a comprehensive learning arena. Overall, different co-ordination practices for work organization among the students were identified.

**Conclusions:**

IPE in nursing homes facilitated the students’ scope from a fragmented approach of the patients towards a relational and collaborative practice that can improve patient care and strengthen understanding of IPE. The study also demonstrated the need for preparatory teamwork training to gain maximum benefit from the experience. Something that can be organized by the education institutions in the form of a stepwise learning module and as an online pre-training course in interprofessional teamwork. Further, focusing on the need for well thought through processes of the activity by the institutions and the timing the practice component in students’ curricula. This could ensure that IPE is experienced more efficient by the students.

**Supplementary Information:**

The online version contains supplementary material available at 10.1186/s12909-021-02867-6.

## Background

Multi-morbidity will increase correspondingly with population ageing, and there will be a growing demand for a comprehensive approach in meeting complex patients’ needs [1–4]. Interprofessional education (IPE) is one way to meet the requirement to manage complex patients and to provide an age-friendly care in multiprofessional teams [5]. However, teaching is still mainly silo orientated [6]. Therefore, the clear desire for increased interprofessional cooperation among health care professionals from policymakers, teachers, researchers and students [7, 8] should be better reflected both in practice and in the educational curricula [9] focusing on the clinical learning environments.

To prepare for an increasingly more complex health care sector, several IPE programs have been implemented within and across universities worldwide [7]. The evidence of IPE refers to strong educational outcomes such as change in attitudes, skills and knowledge, favouring patients’ clinical outcomes [10, 11]. IPE can strengthen students’ own professional role identity, as well as enhance recognition and understanding of other health care professionals’ work [10]. Furthermore, patient-related outcomes can be improved by interprofessional collaboration, e.g. achievement of targeted blood pressure, fewer clinical errors and shorter hospital stays [12].

Studies in a clinical setting focusing on interprofessional teams that include students from other than the medical and nursing professions are sparse [12, 13]. Overall, few studies have applied qualitative or theoretical approaches in addressing the effects of interprofessional learning or students’ experiences of interprofessional collaboration (IPC) and IPE [10, 12–14]. Cox et al. identified a need for IPE to take place in clinical environments [10]. To make IPE relevant for students and potentially impact the health care system, educational activity should not take place in a classroom [10].

The knowledge about multiprofessional students’ experiences from a practice-based IPE orientation in nursing homes for elderly complex patients [15–19] is particularly limited. Nursing homes can create a good learning platform for students to understand all aspects of a patient’s health and especially of those with complex care needs [17, 19]. Therefore, IPE with elderly complex patients could also be a means by which to foster age-friendly care within the healthcare systems [5]. This study aims to describe graduate students’ experiences of working in practice-based interprofessional teams in nursing homes, with patients in the need of complex care. The research question was, “What experiences do students in multiprofessional teams share and obtain from examination of complex patients in nursing homes?”

## Methods and material

A qualitative methodology with a descriptive design was chosen [20, 21]. Focus groups were applied for data collection [20–23]. Focus groups open for group processes and dynamics enabling the participants to respond to statements from other group members, stimulate spontaneous expressive and emotional views, and challenge each other’s opinions about the experience [20, 22]. As the IPE activity is team-based, focus groups could be argued to be a good alternative for the data collection compared to interviews [19–21]. The consolidated criteria for reporting qualitative research (COREQ) were used when writing this paper [24].

### Setting

Since 2016, the University of Oslo (Norway), has included students in a course founded on a collaboration between four faculties and the Nursing Home Agency in Oslo Municipality. It aims at giving students from different health professions the opportunity to learn together in real clinical practice. The course includes students in advanced geriatric nursing, clinical nutrition, dentistry, medicine, pharmacy and psychology who have no previous experience with IPE. The course takes place during the latter part of the students’ training and constitutes an elective part of the curriculum of their curriculum. The main learning outcomes are (1) to gain knowledge about other health care professionals’ focus and approach when examining a complex patient in a nursing home; (2) to perform an interprofessional assessment of a patient; (3) to reflect on the value of interprofessional collaboration in the health care sector (see Additional file 1 for more information about the course).

### Data collection instrument

A semi-structured interview guide with open-ended questions was developed, comprising topics based on the study aim and previous informal project evaluations. The interview guide was concerned with four main questions (1) *How did you experience the organization of the course? 2) How did you solve the task the group got? 3) What have you learned from the course regarding interprofessional learning? 4) How can the course be improved?).* In addition, probing questions were included. Members of the multiprofessional research team, consisting of staff members from advanced geriatric nursing (RH), medicine (EOR, BK) and pharmacy (KS, LM) discussed the guide to ascertain relevance. The guide and the moderation were debriefed after the first focus groups. No major changes were made.

### Sample strategy

All 21 students participating in the course during spring 2018 were invited to participate in the study by e-mail and were provided oral information at a meeting before they enrolled in the nursing home practice component. The students represented advanced geriatric nursing, clinical nutrition, dentistry, medicine, and pharmacy and were all in their final year of the master’s programmes. For practical reasons, psychology students could not participate in the project during the spring 2018.

### Data collection

Four focus group (group size 4–7) sessions were conducted at three different nursing homes after the students’ final presentation of their clinical findings. The focus group interviews were based on the five established interprofessional student teams, except at one nursing home where two teams were interviewed together. All 21 students participated in the study: six students from medicine, six from dentistry, five from pharmacy, two from advanced geriatric nursing, and two from clinical nutrition. Sixteen were females and five were males. Median age was 25 years (range 22–42 years). The interviews lasted for approximately one hour each. We strove for an open discussion environment through warm-up questions. KS moderated two focus groups and assisted in one; BK moderated one and participated as an assistant in two. In addition, RH moderated one group where LM acted as assistant moderator. All group interviews were audio-taped and transcribed verbatim by KS and BK. Participating students received a cinema ticket worth 250 NOK and a light meal during the focus groups.

### Data analysis

An inductive thematic data analysis based on Systematic Text Condensation (STC) was carried out. STC is suitable for cross-case analysis of qualitative data (ibid) based on our descriptive design [20, 21, 25]. Notes from students’ patient case presentations complemented the data analysis. KS, BK, RH, LM and EOR, participated in the initial analysis during which preliminary themes were identified. The analysis began with a process of familiarisation with the transcripts. All researchers read through the data material individually. In a consensus meeting, three preliminary themes were identified (relations, organisation and learning outcomes) for further analysis. See Table 1 for an example of the process. Following this, the five researchers each sorted the transcript of one focus group according to these preliminary themes, i.e. identified meaning units, and coded them. In a second consensus meeting, the initial sorting was discussed and some clarifications were made. Thereafter, KS further organised the material into main themes with descriptions, which was then discussed by the analysis team. Relevant quotes were extracted from the data to illustrate the final themes. The quotes are presented with a student number and their profession with the following abbreviations: AGN = advanced geriatric nursing, CN = clinical nutrition, D = dentistry, M = medicine and PH = pharmacy. Microsoft® Word 2016 was used to manually sort the data.

**Table 1:** An example of how the systematic text condensation was conducted [25]

1. Total impression – from chaos to themes	2. Identifying and sorting meaning units – from themes to codes	3.a) Condensation – from code to meaning^a^	3.b) Synthesising – from codes to descriptions and concepts
Overall impressions from the transcripts were identified, e.g.: The students seemed to experience the IPE-training as meaningful, as they did not seem to know much about each other’s competence in advance. L*earning outcomes* was identified as one of the preliminary themes.	Meaning units from the transcripts were identified, e.g. this quote from a student in clinical nutrition: *“You see how dependent you are on the other professions to find the underlying factors that can influence food intake. I definitely saw the value of having all the professions present. That was very cool. [You understand] how you can use each other on those things.”* The unit of meaning was coded as *new understanding/awareness of each other.*	The units of meaning from each of the code groups were condensed and written into the result section. E.g.: *“For most students, the interprofessional situation was described as new; regarding both collaboration and the ambiguous approach to solving patients’ complex health needs together.”* The themes were redefined and renamed during the process. This example was placed under the theme *Complex patients as learning opportunities - an eye-opener for future IPC.*	Some citations from the transcripts were extracted to illustrate each of the themes. The results were compared to existing literature on the field. The essence of this example was described thusly: *“IPE in nursing homes appears to create a safe forum for students to step out of their uniprofessional approach (and to adopt) other more collaborative practices.”* To ensure a correct interpretation of the data, the transcripts were read through again by one of the researchers.

^a^According to Malterud, this step normally involves writing the condensate in first-person [25]. However, this was not done in our analysis inspired by Malterud. Instead, the text was directly written in third-person

#### Analytical framework

During the final analytical phase, four types of co-ordination practices were found to be helpful in understanding the ways the students approached and performed different collaborative practices during their IPE activities [26]. The four types of coordination practices were derived in a scoping review following reorganization of the Norwegian health care system [26]. Although the coordination practices are revealed from a specific context, the concept has generic features that make it useful to understand broader perspectives such as, for example, IPE. The four coordination practices are^1^:

1. *Relational collaboration*, characterized by closeness in the coordination practice, and a high level of interlocking of the various professions’ various understandings. This may be achieved through many and various physical meetings and negotiations between the actors. Relational collaboration entails a common understanding and recognition of each other’s contributions in the task-solving process.
2. In *Operational closed collaboration* the professionals find it challenging to come to a consensus in their collaboration, and conflicts sometimes arise. This happens even if the professionals may have available physical meeting points and contact between the professions. As a result, the actors never reach a common understanding about the tasks they are expected to collaborate on. In this kind of collaboration climate, they continue to have a differentiated task to solve.
3. *Coordinated delegation* is understood as greater physical distance, but still an interlocking among the professions regarding the integration across the professions’ different understandings. The coordination, closeness, and integration could be done by well-functioning Information and Communications Technology (ICT) systems, written guidelines or via a coordinator.
4. *Split task distribution* is characterized by distance and differentiation where the professions do not have any physical meeting places, and thus, have low mutual knowledge of each other.

### Ethics

The study was carried out in accordance with relevant guidelines and regulations and was approved by the Norwegian Centre for Research Data (reference number: 59948) [27], being the instance responsible for approving research projects processing personal data, and providing data protection services. The respondents gave their written informed consent, and participation was voluntary. Furthermore, the students were informed that they could withdraw their consent at any time without any consequences for their academic grades. The focus group interviews were undertaken by researchers not involved in the formative evaluation of the students. All data information such as focus groups’ recordings and informed consents were stored separately and encrypted in two locked cabinets during the transcription phase, following which they were deleted.

## Results

Three main themes were identified. The first one is called *Complex patients as learning opportunities- an eye-opener for future IPC*. Under this theme, we describe how patients with complex health issues in nursing homes seemed to create a specific learning opportunity for current and future interprofessional collaborations and how students experienced the learning activity. The second theme is labelled *A cobweb of relations*. Our analysis revealed that students who participate in IPE on complex patients encounter multiple relations that they need to manage during the activity. The third theme, named *Structural facilitators for new collective knowledge,* describes important factors for the IPE activity to take place and be perceived by students as efficient*.* The three themes are further worked out in detail in the following three sections.

### Complex patients as learning opportunities- an eye-opener for future IPC

The students expressed that nursing homes were well suited for interprofessional training because most of the patients are admitted on a permanent basis and have complex needs. However, some students felt that the situation was unrealistic, as interprofessional collaboration is not the standard model in nursing homes in Norway. Some therefore questioned the learning experience as they doubted this collaboration would be possible to practice afterwards in real life. Still, all students appreciated the value of interprofessional collaboration on complex patients and wanted more of it, as expressed by one pharmacy student:

**Group A**

*Student 21 (PH): I didn’t even know you [medical student] were supposed to do that examination. It was quite fun to watch you do it, and to see how it helped. Usually we just talk to the doctors over the phone.*

For most students, the interprofessional situation was described as new; regarding both collaboration and the approach to solve patients’ complex health needs together. The students reflected about how the interprofessional student team could help with the patients’ complex health needs. A few students mentioned that they felt a new kind of security with regard to the patient’s health, as they took decisions as a group with various professional backgrounds. Some students nevertheless mentioned and experienced a tension when prioritising the different suggestions for a patient’s complex health care needs. In a way, they described that they perceived such prioritising as a challenge, but also referred to this as an incentive for learning as it broadened their perspectives on a health care problem.

A few students declared that they were used to being assigned a patient’s specific problem, and to outlining a plan for treatment and care. In this course, the student teams encountered patients with no “predefined problems” to take care of, and this was described as a new and challenging experience for them. In some of the cases where the students were unable to find ways to describe or define the problems they encountered, they suggested that these should be characterised as “psychiatric problems” and refrained from further investigation. Nevertheless, all groups experienced patients with varying degrees of psychological or psychosocial problems, group B expressed:

**Group B**

*Student 13 (AGN): Deviant behaviour in nursing homes is not an unusual phenomenon. You must be able to understand what’s going on.*

*Student 16 (M): [ … ] We discussed the patient with the other group [during the account of the group project], and then someone suggested that it might be psychiatry. And then it stopped …*

*Student 13 (AGN): I mean like “where does one start”?*

*Student 16 (M): You don’t have anything to come up with. What could you do about it?*

Therefore, the students reflected about the difficulties in encountering, confronting and finding solutions to such problems, and during the semester during which the psychology students were unable to attend, many of the students expressed that they would have preferred to have a psychology student on their team to help patients with these needs.

The students explained the learning that took place through observing others examine patients and paying attention to the way they asked questions, and subsequently through the joint discussions about the patient cases. For example, one student expressed being surprised by how much important information other professionals could gain in a very short amount of time. Another student described his/her experience of how dependent the students were on each other to solve complex health care problems. The students stated that their personal experience of interprofessional collaboration was a strong factor in lowering the threshold for contact and actively using each other as resources in future work. The students also mentioned how they had explored their professional selves. This was said to strengthen their own learning. They expressed that they learnt to trust their own knowledge, skills and abilities as representatives for their respective professions and that they thereby perceived professional development.

**Group C**

*Student 8 (D): You get a wider perspective when you see the others do their examinations and evaluations. You kind of get the whole picture.*

*Student 6 (D): I also start to think about other things. For instance, if you see that a patient is on certain medications, you think “what did the doctor think? Why is the patient on these medications?”. But now I understand that it’s not so easy to deprescribe certain medications. The patient is not always willing to do so. You don’t think about that when you work separately. You think you know best, most of the time. But now we don’t, so it was fine to have that experience as well.*

### A cobweb of relations

The students described their relations with the patients in a very respectful way, always aiming to place the patient at the centre of the team’s attention. The students mentioned ways of approaching the patients with non-invasive examinations and by offering breaks when the patient was tired. During the focus groups, many students reflected that, as a group, they influenced the patient encounter in a slightly different way compared to the uniprofessional approach; an even more imbalanced power situation could arise as the students outnumbered the patient. Some thought this could hamper patients’ willingness to share their problems, beyond the fact that the examination took more time and had a tendency to tire the patient. Nevertheless, a couple of students stated that the patients appeared to have enjoyed the experiences, felt safe and shared their problems willingly. Several students stressed that it was important and a strong motivating factor that the project was relevant for the patient and not merely for the students’ learning. In addition, they stressed the significance of communicating their conclusions to the patient and to the staff at the nursing home. Several students expressed having felt nervous and insecure about their knowledge, role and abilities before they met the other group members. Most students regarded the informal information meeting ahead of the practical exercise as very positive and as a “relational ice-breaker”.

**Group A**

*Student 18 (CN): I thought it was nice to be prepared for a situation like this, because you are supposed to speak up. I must say that I felt a bit nervous, but it might be because I don’t have so much clinical experience yet. It was very nice, though! We met a nice group. I felt like I could say what came to mind without being afraid that what I said wouldn’t be “academic enough”. I dreaded taking a measurement “here” in front of the medical student because I couldn’t say the right anatomic names [ … ].*

Some students described the importance of individual qualities to form successful student teams, such as non-judgmental openness to others’ ideas and a willingness to listen and show interest in the viewpoints of the other team members. Having a common reference, such as medications for the dentist, doctor and the pharmacists, appeared to be a useful starting point for the relationship, facilitating a common understanding. The students said the awareness of expectations about each other contributed to building these new relations. None of the students mentioned a need for training in interprofessional collaboration in advance of the course even when asked directly during the focus groups.

The students pointed out the importance of building a good relationship with the nursing home’s staff members (doctors, nurses and nurse assistants) to enhance the students’ possibilities to improve patient outcomes. They emphasised and appreciated the knowledge shared by the permanent staff, thus helping them to gain a better understanding of the patient’s history and daily situation. To ensure that their work was transferred back to the care of the patient, they saw the need for staff to be present in the patient case presentations, as well as in the initial patient information-gathering phase.

Some students described the importance of forming good relationships with the educators, who could provide security and support in their new role in the interprofessional teams. For example, the educators helped to confirm and discuss their findings, and they provided feedback in relation to the level of details of the examinations. The students also mentioned the safe learning environments during the patient case presentations. They referred to this as a positive learning experience, because the educators were interested in what they had found out about the patient, rather than in examining the students.

### Structural facilitators for new collective knowledge

The participants pointed out that the organisation and collaboration in the student teams played an essential role in utilising the additional learning effects of the interprofessional student team on complex patients. Both the students’ own organisation of their teamwork, and how they experienced the support of the administration behind, and available time for IPE were described as central in creating a well-functioning IPE activity.

Although it was perceived that the structure for facilitating interprofessional approaches and understanding were in place (from the perspective of the educators), our findings show variation in the amount of time students cooperated with one another. Some stated that they had cooperated a lot and had discussed the patient together. They cited this experience as very valuable for their learning experience. On the other hand, some students mentioned that they had not discussed their clinical findings with the other group members at all. In addition, there were groups who cooperated to varying degrees between these endpoints. In the focus group discussions, students admitted having had less close collaboration and not having finalised their patient care plan together; instead, they said that they had worked by themselves individually to solve the problems most relevant for their profession, e.g. the dentist had solely focused on dental health. In one group where there were two team members from the same professions, the students said they had teamed up with colleagues from their own profession without engaging in the new interprofessional team. The variation in the groups’ collaboration is illustrated by these quotations:

**Group D**

*Student 5 (M): Well, we medical students went together (before meeting the patient), and also the pharmacy student, and looked at it alone. The dentistry students looked at what they planned to ask about. We grouped [uniprofessionally] in a way. That’s basically what you have to do too know what to ask for from your own field of expertise.*

*Student 4 (M): And then we worked by ourselves to look through the information we got from the anamnesis. That has been kept separate, and we have not coordinated any information. So, the report meeting was actually the first time we were able to listen to findings and solutions from each other.*

*Student 5 (M): It would have taken a long time to arrange a meeting for all of us[ … ] .*

***Group B***

*Student 13 (AGN): Sitting in the same room is a huge advantage. When a spontaneous thought comes to you, you can bounce your ideas off one other and ask the person who probably knows the most. You can’t do that reading documents or when you are at home working with the document [this group worked together on a document online], where you just put in your own notes. It is useful to be in the same place.*

*Student 17 (CN): You get to “challenge your view”, because it broadens your perspective, but also because you get another point of view.*

The students discussed the availability of accessible patient information prior to the patient assessment and examination. On one hand, more substantial information could rationalise their examinations. On the other hand, some students expressed that too much information may limit their interprofessional learning in gathering important data.

**Group C**

*Student 10 (M): We didn’t get the whole picture. And I wasn’t sure how much of the picture we were supposed to get. Was it intended that we should not be prejudiced? Or were we supposed to perform the examination so thoroughly that we also understood how it has been (the status of the patient’s status over the period of the past weeks or months)?*

*Student 12 (AGN): And we figured out that not necessarily everything the patient said was correct. I think it was good to know what had been done in advance, so we could prioritise which examinations we should do within the time constraints.*

Time was an issue for the students both in relation to students’ professional maturity, i.e. when in the curriculum the interprofessional workplace training should be scheduled, and in relation to the time needed to carry out the training. As an example, the students discussed what might constitute sufficient time between meeting the patient and the patient presentations, what was a suitable time in the curriculum and, how much time they needed for examining and building relations with the patient. Other examples were time, for the team to have enough access to the patient and for processing the findings and searching for additional information. Afterwards one group reflected on the possibility that time might have been saved if the participants had engaged each other more in the patient interview. The students wanted training that was more condensed and adjusted to their individual time schedules.

## Discussion

This study aimed at increasing the knowledge about students’ experiences with IPE in a setting including patients with complex care needs in nursing homes. The major finding is that students are challenged on many levels when collaborating on complex patients. For example, when examining patients with no predefined problems, the students were forced to look at the patients from many professional angles. They experienced that they had to build relations with several participants in the learning activity to solve the patients’ care needs. From the discussions, the students seemed to develop patient relations as a team and as individuals that were dependent on previous team members’ actions. The various ways of collaborating as teams challenged their normal way of working as individual professionals. Our practice-based IPE activity appears to be in line with some of the components of an age-friendly care, and could be a means to support a better healthcare system for the elderly patients [5]. In a practice-based IPE activity in nursing homes, the 4 M’s framework (medication, mentation, mobility and what matters most) could be carried out by establishing multiprofessional student teams [5]. Furthermore, by incorporating a psychologist and physiotherapist in the team may strengthen the activity even more. On the other hand, the patient should be protected from having too many students present simultaneously as this can be exhausting. Our students also expressed that there were possibly too many students to benefit optimally from the IPE. Thus, future studies should investigate ideal group size and combination of students to achieve targeted learning outcomes. Our findings are in line with other studies [17, 19] also pointing out nursing homes as appropriate arenas for a practice-based IPE activity. These arenas provide an opportunity for students to experience very complex patients’ situations during their education. Although this was challenging and sometimes frustrating, the opportunity they had to discuss difficulties with each other and the faculty (educators) enhanced their understanding of the patients’ needs and the advantages of collaboration.

IPE in nursing homes turned out to be a challenging, but safe environment enabling students to distance themselves from a uniprofessional approach and discover other, more collaborative practices. Our findings suggest that the approaches used by the students to structure their collaboration are important for their total learning experience targeting complex patient needs (see the theme *Structural facilitators for new collective knowledge* above, and Table 2)*.* The groups had different approaches towards solving the patient case, clearly reflected in Vik’s four types of co-ordination practices (see method section and Table 2) [26]. In particular, the *relational collaboration* become evident in IPE in nursing homes [26]. We argue that IPE in nursing homes expanded most students’ educational horizon from having a *split task* approach towards patients to experiencing a relational and collaborative practice [26].

**Table 2:** Vik’s typology and examples of how some of these approaches were evident in the focus group data [26]

*Co-ordination practice*	Examples from focus group data
*Relational collaboration*	*Group B*
	*Student 13 (AGN): It is a huge advantage when we sit in the same room. When you spontaneously get a thought, you can bounce your idea off each other and ask the one that probably knows the most. You can’t do that reading documents or when you are at home and working with the document [this group worked together on a document online], where you just put in your own notes. It is useful to be in the same place.*
	*Student 17 (CN): You get to “challenge your view”, both because it broadens your perspective, but also because you get another point of view.*
*Operational closed collaboration*	Group D
	*Student 5 (M): Well, we medical students went together (before meeting the patient), and also the pharmacy student, and looked at it alone. The dentistry students looked at what they planned to ask about. We grouped [uniprofessionally] in a way. That’s almost what you must do (so as to) know what to ask for from your own field of expertise.*
	*Student 4 (M): And then we worked by ourselves to look through the information we got from the anamnesis. That was separate, and we have not coordinated any information. So, the report meeting was actually the first time we got to listen to findings and solutions from each other.*
	*Student 5 (M): It would have taken a long time to arrange a meeting for all of us […*].
*Coordinated delegation*	
	*Group B started having physical meetings together and thereafter continued working on a document online. See quotation above. The group mainly favoured working relational, but had elements of coordinated delegation.*
*Split task distribution*	*Group D*
	*Student 4 (M): I feel ambivalent. The learning outcome would be to know that I feel secure enough to ask the dentists about things. But the dentists are not [normally] present at the nursing home. So, the situation is not realistic. They sit in their offices, and I have got a verification that they are not present in the nursing homes, which is a problem.*
	*Researcher: You think this training was a bit unrealistic?*
	*Student 4 (M): Yes, a bit unrealistic! Since neither the dentists nor the pharmacists usually are in the nursing homes, we somewhat introduce these experts [in the team]. And now I can see the benefits of obtaining expertise when examining a patient. It is important with an interprofessional approach, but I am not sure of how I can use this further, because I guess that they will not be there in that way [in the real world].*

In the theme *Structural facilitators for new collective knowledge,* we describe groups with a focus on establishing physical meetings throughout the whole process and having discussions with a view to sharing knowledge (Table 2). Students expressed that they had achieved a different collective knowledge, i.e. a deeper understanding of the patient and each other, compared to groups that did not meet more often than scheduled. Therefore, some groups appeared to have engaged more in *relational collaboration* practice as described by Vik [26].

On the other hand, some of the teams in our course did not fully engaged in the interprofessional collaboration (Table 2). They presented uniprofessional suggestions for the complex patients’ health problems without building on their potential strengths as an interprofessional group. Such a practice is more influenced by an *operationally closed collaboration* [26]. For example, in our course, the students had the opportunity to meet each other during the course work, but not everyone took advantage of these opportunities. It was apparent in our results that in such teams, the students never reach a common understanding of the task and each other’s role, despite provisions for various physical meeting points.

Practice-based IPE in nursing homes appears to be helpful for understanding the complexity of older adult care and gaining an appreciation of a collaborative approach, but students may need some preparatory teamwork training to gain maximum benefit from the experience. In contrast to the students who did not see a need for training when asked, our findings indicate the importance of the physical and social organisation of the teams to achieve maximum learning outcomes, and potentially also improving clinical outcomes for the patients. Therefore, to achieve a successful *relational collaborative* IPE practice, pre-training in teamwork might be needed, i.e. preliminary training introducing theoretical knowledge about the other team members, information about how to create successful interprofessional teams, simulation exercises and a discussion about how physical arenas, in addition to online software, can create such platforms for collaboration. Pre-training e.g. in a stepwise learning module, investing some time beforehand and introducing tools by which to evaluate their ongoing collaborative activity are important training components. Moreover, improvements of several administrative factors need to be considered in ensuring a *relational collaborative* practice experience [14, 26, 28]. In our study, this is exemplified by the students’ wish for an effective patient meeting in terms of time efficiency, the need for including only one participant per profession in the group, and a better aligned time schedule. Hean et al. [14] argue that social aspects of learning, in particular relationship building between participants, distinguish interprofessional learning from uniprofessional learning. Social aspects of learning by the conflicts or negotiations between the students’ different competences, roles and understanding will then help to create a mutual knowledge (or mutual understanding of each other’s competences). That should form a good starting point for further interaction such as by *relational collaborative* practices and/or a *coordinated delegation* practice which is understood as physical distance, but still a mutual acceptance of the professions’ different understanding. The integration could be done, for example, by well-functioning Information and Communications Technology (ICT) systems [10, 14, 26].

The students expressed that the healthcare sector today is not characterised by interprofessional approaches and is more oriented towards distanciation and differentiation, i.e. *split-task distribution* meaning that collaboration is characterised by distance and differentiation [26]. When starting their professional lives, students who have experienced a practice of *relational collaboration* during their education might lower the threshold for conferring with other professionals. By knowing each other’s limitations and strengths, for example, physical distancing will not be equivalent to a uniprofessional approach, and a *coordinated delegation* might occur [26]. This meets the rationale for IPE, that learning together enhances future working together and improves factors such as leadership, collaboration and communication between healthcare professionals [11]. Developing these skills in healthcare students is justified by taking these three factors into account.

### Methodological discussion

One limitation of this study might be the fact that two moderators were involved, as two focus groups were operational simultaneously in the same nursing home. Immediately after the first two focus groups, a de-briefing session was held with the moderators and co-moderators, to discuss the interview guide. Rather than being a limitation, we experienced that the use of two moderator teams actually enriched the interviews. One of the strengths of this study is the interprofessional research team working together, giving a wider perspective on the study design, analysis and interpretation of the empirical data collected. The ambition in this project was related to the idea of creating dialogue, expanding the students’ perspectives, and including a multi-disciplinary approach. During the interviews, we were aware of this and challenged the students to bring out different experiences.

RH and LM, who conducted one of the focus group interviews, had previously been involved in the course as educators, but they did not play a major role at the time of the data collection. KS and BK had no previous connection to the project and were enrolled to be an independent part of the data collection and analysis team. By using the already established student teams, we might have missed some negative experiences from the teamwork. On the other hand, the students seemed to be confident and able to express challenges regarding IPE.

It is questionable as to whether saturation was achieved; however, the material provided us with thick and rich information, which contributed new insights about the students’ experiences with practice-based IPE. In assessing saturation, the five factors that were identified by Malterud et al. [29] as important for information power are discussed. The *study aim* was narrow, which requires fewer participants than a broader aim and reflects a large sample consisting of 21 participants including a minimum of two from each profession. Regarding students, the *sample specificity* was medium – the students had experience from the activity; however, the course is restricted to only two days of IPE activity. An *established theory* was not included in the study. We judged the *quality of the dialogue* as high (see the reasoning above about), and hence increasing the information power. In our *analysis a* cross case was used, which requires more participants. As stated above the sample included 21 participants, which can be claimed to be sufficient.

## Conclusions

Interprofessional education in nursing homes entails challenges to and possibilities for developing the healthcare services and for supporting age-friendly care for elderly patients with complex care needs. The training in nursing homes broadened students’ horizons by abandoning a fragmented approach towards the patients and adopting a relational and collaborative practice that can improve patient care. Practice-based IPE in nursing homes appears to be helpful for understanding the complexity of older adult care and gaining an appreciation of a collaborative approach. Nevertheless, students may need some preparatory teamwork training to benefit optimally from the experience. We suggest that educators arrange practice-based IPE activity at an early stage, to foster more collaborative practices e.g. by a stepwise learning module, simulation exercises or an online theoretical pre-training course in interprofessional teamwork. Furthermore, there is a need for the institutions to focus on well thought-out processes for the activity as well as the timing of the practice component in the students’ curriculum. This might ensure that the students perceive IPE as more efficient.

## Supplementary Information

**Additional file 1.** Description of the course.

## Data Availability

The datasets used and/or analysed during the current study are available from the corresponding author by reasoned request.

## Abbreviations

COREQ: consolidated criteria for reporting qualitative research
IPC: Interprofessional collaboration
IPE: Interprofessional education
WHO: World Health Organisation

## Footnotes

1 The translations from Norwegian are those of the authors of this paper and not Vik’s.
