# Supplementary material for: Interprofessional education on complex patients in nursing homes: a focus group study
Source: BMC Med Educ. 2021 Sep 24;21:504. doi: 10.1186/s12909-021-02867-6 (PMC8464088; doi:10.1186/s12909-021-02867-6)
Supplement: Supplementary file 1 — Additional file 1. Description of the course. [file 12909_2021_2867_MOESM1_ESM.docx]

**Appendix 1. Description of the course.**

In the spring of 2018, the course was conducted as a pilot project for the fourth time. A total of 40 students have participated in the four pilot semesters. Before the clinical practice, the students participated in an evening meeting, the purpose of which was to provide information about the course. Furthermore, the students had the opportunity to socialize in their respective groups consisting of students from each of the six professional educations. The students received a practice booklet encompassing learning goals, templates for each disciplinary examination check-lists, and a two-days’ time schedule. On day one, they convened at the nursing home, where they planned and conducted an interview and examination of a nursing home patient with complex care needs, such as diabetes, heart failure, respiratory dysfunction and psychiatric disorders. An important feature of the training was that the students were to collaborate in the planning of the examinations and observe one another during the consultation with the patient, including observing and learning more about a pharmacy student performing a medication review or a dentist examining the dental status of the patient.

After the examination, the students discussed their findings and observations, and prepared a joint report and a plan for further interventions for the patient. On day two, one week after the patient assessment, the groups gathered again and gave an oral presentation of the action plan (“patient-case presentation”) for the healthcare staff responsible for the patient at the nursing home, their student colleagues and the educators from the University. One third of this session was dedicated to responses and discussion with the audience. Finally, the group submitted their written report to the educators, one part reporting about the action plan for the patient and one part encompassing a description of the group process.
